# Development of predictive and evaluation models for the retro reflectivity performance of pavement lane markings

**DOI:** 10.12688/f1000research.169127.2

**Published:** 2025-10-06

**Authors:** Dowan Kim

**Affiliations:** 1 Road & Airport Dept., Kunhwa Engineering & Consulting, Seoul, 70222, South Korea

**Keywords:** Retroreflectivity, GRG algorithm, Seoul Pavement Index (SPI), Seoul Road Markings Index (SRMI), Pavement Marking

## Abstract

**Background:**

The functions of pavement-markings are to safely and smoothly guide drivers and vehicles along a road-lane, alert drivers not to leave the lane, and provide traffic information. These functions are directly related to the safety and economy of passengers and vehicles on roads. The visibility of pavement-markings is linked to surface damage during the daytime and is significantly affected by luminance at night. Therefore, damage and luminance should be considered key-factors for maintenance and should be measured in detail. In Seoul, mobile equipment such as image-scanners and damage-detectors has been used to measure damage conditions, and a luminance measurement vehicle has been introduced to observe reflected light intensity from road-markings. The mobile measurement methods are respectively utilized due to their objective difference of the measurement. However, measurement discrepancies in temporal, locational, and environmental conditions can lead to considerable errors. Moreover, a standardized maintenance index is currently lacking for planning repair work in Seoul. In that regards, one objective of this research was to develop a retroreflectivity prediction model using a vehicle that detects pavement and marking damage.

**Method:**

The model enables the prediction of retroreflectivity without additional measurement equipment. Another objective was to develop degradation models based on the age of pavement-markings and to evaluate their performance for maintenance and budget planning.

**Results:**

As a result, a regression model was determined to be the optimal predictive model for retroreflectivity. A logarithmic model was selected to predict retroreflectivity degradation. Additionally, the Seoul-Road-Marking-Index was developed.

**Conclusion:**

The developed models are expected to assist in establishing maintenance plans, formulating budgets, and determining repair work, as well as developing relevant regulations and standards in Seoul.

## Introduction

Pavement markings that guide vehicles to safely drive within a lane and prevent departure from it are one of the basic facilities for transmitting various kinds of information to drivers. This significantly affects the safety of vehicles, passengers, and other road users. If these markings do not function as intended, serious accidents may occur. Therefore, maintenance and repairs should be conducted thoroughly. Luminance and physical damage are major safety factors that affect visibility at night and during the day, respectively ( Lee and Cho, 2023). Thus, maintenance and repair work should be implemented based on damage severity and luminance. Performance measurement of reflected luminance from pavement markings has been performed using portable equipment or a mobile measurement vehicle. The method for measuring luminance performance involves observing the reflected light intensity generated by light equipment attached to the measurement vehicle, which interacts with beads embedded in the pavement marking. In the Republic of Korea, portable equipment is commonly used for performance measurement of reflected luminance. However, this method is underutilized due to safety concerns for measurement workers and civil complaints arising from traffic control and congestion in high-traffic areas (e.g., downtown areas, expressways, and highways). Therefore, a mobile measurement vehicle is required to ensure the safety and effectiveness of the measurement process. This method is specifically utilized by road management offices that possess such vehicles. Damage conditions can be measured using another vehicle. In the Republic of Korea, mobile measurement vehicles equipped with image scanning devices, damage detectors, and GPS are generally utilized to assess damage to pavements and pavement markings. These vehicles can commonly measure pavement conditions such as roughness, plastic deformation, fatigue cracking, and potholes, as well as damage to pavement markings. However, these methods have limitations. When retroreflectivity of pavement markings is measured, various factors—such as time of measurement, weather, reflectance from nearby facilities, and road conditions—can affect the measurement and analysis results ( Pike et al., 2007; Zhao et al., 2023). Moreover, differences in light range and intensity can cause significant measurement variation ( Babić et al., 2022), even when using the same equipment under seemingly identical conditions. To reduce errors in measurements obtained using a mobile measurement vehicle, portable equipment can be utilized manually. However, because human-operated measurements cannot be applied to all pavement markings, measurement reliability may be compromised. Additionally, worker accidents, civil complaints, and road disruptions due to traffic congestion may occur. Although another damage-measurement vehicle can effectively assess pavement and road marking conditions, it may cause safety issues and traffic congestion, as it occupies two lanes while measuring lane markings between them using centrally installed equipment. Moreover, analysis results may include serious time-based and locational errors because the inspection methods for measuring damage and retroreflectivity of pavement markings are conducted separately.

In this regard, the objective of this study is to develop a predictive model for the retroreflectivity of pavement markings by considering the variability introduced by the dual-inspection methods and leveraging the advantages of using a pavement damage measurement vehicle that can easily assess road marking conditions. To achieve this, variables obtained from pavement condition measurements, the damage status of pavement lane markings, and Average Annual Daily Traffic (AADT) were used to develop a retroreflectivity prediction model. In addition, a performance degradation model of retroreflectivity was developed based on the age of pavement markings, using records from completion inspections of marking work. Based on these developed models, an evaluation model for pavement markings was created. When the damage to pavement and road markings is measured, the retroreflectivity degradation prediction model can be applied, and the developed Seoul Road Marking Index (SRMI) can be used. Furthermore, the SRMI model was developed to evaluate performance, determine the need for maintenance work, and manage pavement markings in Seoul City. To achieve the objectives of this study, damage and retroreflectivity measurements were performed concurrently. Model optimization was conducted using the Generalized Reduced Gradient (GRG) algorithm to minimize the Root Mean Squared Error (RMSE) between measured and predicted values.

## Investigation of road markings, pavement condition, and traffic volume

**Figure 1.**
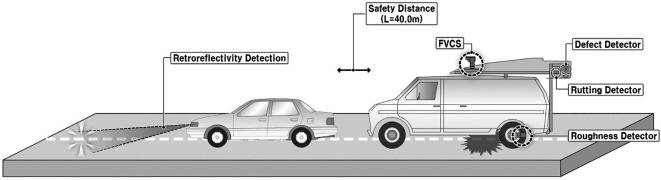
Pavement and Road Marking Condition Investigation Configuration. This figure illustrates a schematic configuration of the investigation conducted in this research to assess the condition of pavement markings and pavement surfaces.

**Figure 2.**
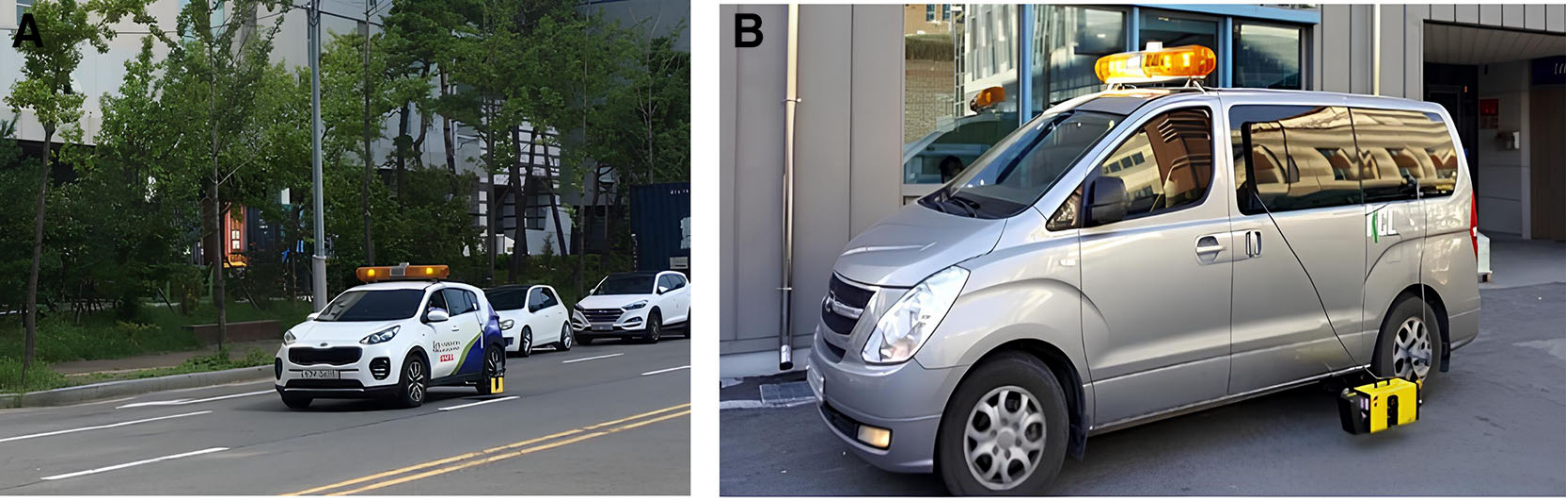
Retroreflectivity (left) and Pavement Condition (right) Measurement Vehicles. The image on the left shows the vehicle used by the Seoul Metropolitan Government to evaluate the condition of pavement markings, while the image on the right shows the vehicle utilized for pavement surface condition.

The major objective of this research was to develop a retroreflectivity prediction model by utilizing damage inspection results. Measurements for damage inspection and retroreflectivity were concurrently performed. Therefore, a vehicle for retroreflectivity measurement was arranged in front of another vehicle to detect the pavement and road-marking conditions. The measurement configuration and vehicles used are shown in Figures 1 and 2.

The measurement implementation focused on 31 sections of eight city roads and pavement lane markings between the first and second lanes in Seoul City. A safe distance between the measurement vehicles of L = 40 m was secured using a Front Vehicle Camera System (FVCS) at all measurement times. The measured variables gathered using these methods include pavement conditions –plastic deformation, fatigue cracking ratio, International Roughness Index (IRI), and Seoul Pavement Index (SPI)– and pavement lane marking conditions –measured road marking area, damage area, damage ratio, and retroreflectivity. The traffic volume information of AADT was collected from the national information sharing system of the Transport Operation & Information System (TOPIS).

In the Republic of Korea, the National Highway Pavement Condition Index (NHPCI) is used for national roads, whereas the Highway Pavement Condition Index (HPCI) and Pavement Condition Index (PCI) are used for expressways and urban, rural, and airport roads. Although the detailed index values of the regression model quantifying the pavement conditions are different, the three distresses used to assess the pavement conditions are essentially the same: cracks, plastic deformation, and IRI. On the other hand, the Seoul Metropolitan Government has introduced a Pavement Management System (PMS) program to efficiently carry out road pavement maintenance, which covers approximately 8,172 km, and evaluates the pavement status according to its own pavement condition index, the SPI.

The SPI is expressed in a 0–10 point system, with 10 points representing ideal road conditions and 0 representing completely deteriorated roads. The individual SPI is an 10point-based representation of the cracking rate (%), plastic deformation (mm), and IRI (m/km) of the road, where the initial SPI on new road pavement is approximately 8.0–9.0. Table 1 shows the standard for repair, and the evaluation of individual distresses in Seoul City. As shown in the table, the SPI is determined by calculating each individual distress index. These individual distress levels are then combined to obtain the total Pavement Damage Index (PDI) to objectively evaluate the pavement condition. The SPI is then determined using the PDI, as explained in the table footnotes. The overall SPI calculation includes a correction function for traffic volume to compensate for the shortcomings of not being able to secure an accurate traffic volume estimation according to the Seoul Metropolitan Government’s standard. The actual repair standard of SPI = 6.0 indicates when road pavement maintenance is required due to a combination of the three distress indices. The correction functions and their coefficients are listed in Table 2.

**Table 1.** Individual Distress Indices Used by Seoul City.

Defect	Equation		Maintenance criteria
${\mathrm{SPI}}_{\text{Crack}}$	10-(1.667* ${\mathrm{CR}}^{0.38}$ )		10%
${\mathrm{SPI}}_{\text{Rutting}}$	10-(0.267*RD)		15 mm
${\mathrm{SPI}}_{\mathrm{IRI}}$	Urban expressway	10-(0.8*IRI)	5 m/km
	Arterial road	10-(0.727*IRI)	5.5 m/km
	Minor artery road	10-(0.667*IRI)	6 m/km

Note: SPI=10−PDI , $\mathrm{PDI}={\left[{\left(10-{\mathrm{SPI}}_{\text{Crack}}\right)}^{5}+{\left(10-{\mathrm{SPI}}_{\text{Rutting}}\right)}^{5}+{\left(10-{\mathrm{SPI}}_{\mathrm{IRI}}\right)}^{5}\right]}^{\frac{1}{5}}$ .

This table outlines the criteria and functions used to evaluate the Seoul Pavement Index (SPI) currently implemented by the Seoul Metropolitan Government.

**Table 2.** SPI coefficients with Road and Maintenance Conditions.

Road coefficient ( $K_{1}$ )			
Road factor	Urban expressway	Arterial road	Minor artery road
$K_{1}$	0.32	0.11	0
Maintenance scale coefficient ( $K_{2}$ )			
Maintenance scale factor	0.5 km below	0.5~1.0 km	More than 1 km
$K_{2}$	1.0	0.990	0.980
${\mathrm{SPI}}^{*}=\left(\mathrm{SPI}-K_{1}\right)\;K_{2}$			

This table shows the SPI coefficients currently used by the Seoul Metropolitan Government for pavement maintenance and management.

A digital image processing method was applied to determine the damaged area and ratio of the pavement markings. The Mask R-CNN algorithm, an Artificial Intelligence (AI) algorithm, was utilized in this study. The Mask R-CNN algorithm is an extension of the Faster R-CNN that simultaneously applies instance segmentation, which distinguishes the boundaries of each object after object detection and classification in the image. In addition, it masks objects according to their shapes based on the bounding box from the Faster R-CNN. The overall process of the Mask R-CNN comprises convolutions, subsampling, and connected layers. In a previous study, an automatic analysis method using Mask R-CNN was developed to locate road surface markings and accurately detect road surface marking boundaries ( Tran et al., 2020). Several studies have used Mask R-CNNs to detect objects on pavement surfaces ( Shaodan et al., 2019; Shi et al., 2019; and Tran et al., 2022). Thus, Mask R-CNN is a widely used method for detecting objects and separating them from the background without difficulty in classification and segmentation. However, in some cases, the object cannot be recognized because of several factors that affect the image. This could be due to differences in lighting, survey speed, and resolution. Several techniques have been used to address this issue. To obtain the information that an image contains, it is necessary to separate the objects it contains; one of the simplest methods is binarization. Binarization is the process of transforming data into two binary units, 0 and 1, to classify objects and backgrounds. After converting the original image to a grayscale image and effectively separating the background and objects by applying a threshold value, the entire image was used for further image processing. If the pixel value is greater than the threshold value, the pixel value at the same location in the resulting image will be white (255). If the pixel value is less than the threshold value, it will appear black (0), resulting in a binary image. The binarization technique has already been used to detect pavement surface objects such as distress ( Gopalakrishnan et al., 2017; and Otsu, 1979).

**Figure 3.**
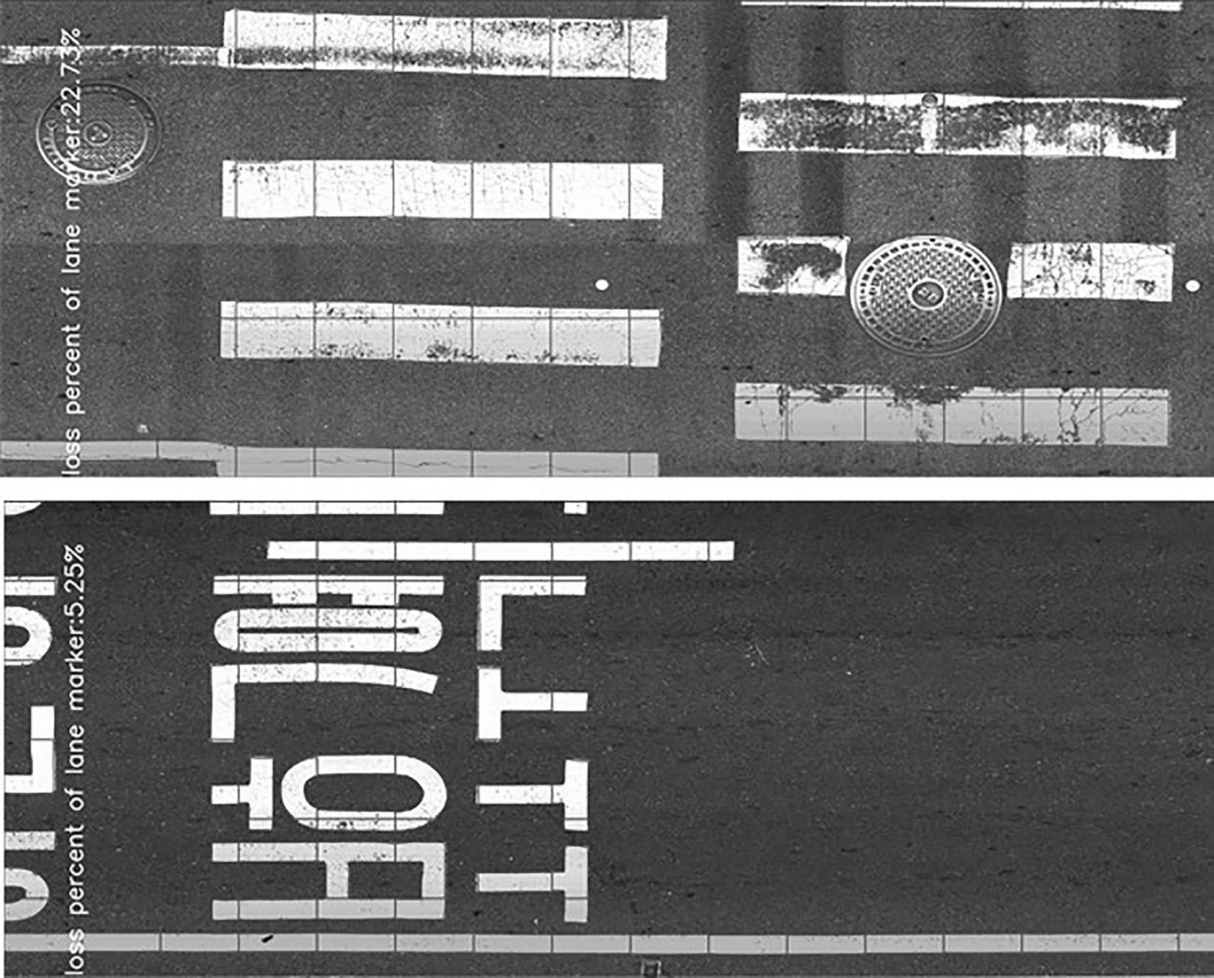
Road Marking Condition Measurement Results. Using digital image processing techniques, parameters such as the defect ratio and measured area of pavement markings can be derived from the imagery collected in this study. This figure presents a portion of the analysis results.

In this study, a combination of current techniques for object detection was applied. To determine the lane marker, lane marker area, and lane marker deteriorated area, a Mask R-CNN was first applied and then binarized. Using this step-by-step process, the lane marker can be isolated from the pavement and the deteriorated area can be determined. The results of this analysis are shown in Figure 3.

Retroreflectivity can be variously expressed, and the general expression is to note the coefficient of the retroreflected luminance $R_{L}$ . It was defined by the American Society for Testing and Materials (ASTM) as the proportion of the observed luminance L on the surface per illuminance perpendicular to the surface $E_{L}$ . This can be expressed as shown in (1).

$$R_{L}=\frac{L}{E_{L}}=\frac{\mathrm{mcd}}{\mathrm{Lux}\times m^{2}}$$ (1)

Mobile measurement equipment can investigate pavement markings based on a unit area of 1 m × 1 m and has a measuring range for the coefficient of retroreflected luminance of 40–2,000 mcd/lux·m ^2^. The sensor installed on the left side of the measurement vehicle was set up to focus on the measurement of pavement lane markings. The data collected from these measurements are presented in Table 3.

**Table 3.** Investigation Results from Data Collection.

Road section	AADT	Pavement				Road markings		
		Crack (%)	Rutting (mm)	IRI (m/km)	SPI	Defect Ratio (%)	Defect Area (mm ^2^)	$R_{L}$
S-1	21,508	19.62	9.33	7.06	2.13	24.35	209,421	91
S-2	21,723	7.65	7.12	1.79	4.77	2.86	20,145	412
S-3	14,994	4.29	5.18	2.72	5.70	6.45	49,770	96
S-4	14,994	1.20	6.53	2.54	6.93	6.81	77,154	140
S-5	14,994	3.39	10.53	6.88	3.82	18.54	120,476	118
W-1	34,693	0.00	3.31	1.21	8.93	1.35	8,795	128
W-2	14,671	0.00	3.98	1.80	8.48	0.61	4,936	112
YA	14,424	0.00	5.93	5.00	6.02	6.87	55,515	75
Y-1	33,910	13.05	12.41	6.24	2.96	11.32	55,682	273
Y-2	34,249	5.85	8.79	4.01	4.68	6.79	41,920	332
E-1	18,970	0.00	3.33	3.02	7.88	9.32	64,914	256
E-2	19,160	0.00	3.18	2.60	7.88	7.14	38,317	315
T-1	20,628	0.00	2.77	2.16	8.26	1.43	46,237	186
T-2	20,834	0.00	3.31	1.73	8.49	0.38	5,948	411
T-3	20,628	0.00	2.71	2.07	8.35	1.51	48,152	158
T-4	20,628	18.96	6.46	2.46	2.63	9.61	80,658	159
T-5	20,834	0.00	2.77	2.52	7.88	0.58	8,999	411
I-1	20,519	3.87	6.99	8.34	3.72	2.80	49,138	113
I-2	20,541	1.23	15.38	9.43	2.63	31.13	318,091	296
I-3	20,541	7.59	15.05	8.31	1.77	44.68	681,021	321
I-4	20,541	0.00	8.39	6.47	5.64	4.76	77,558	326
I-5	20,541	4.13	13.50	3.39	5.49	71.57	846,201	287
I-6	20,747	0.00	7.81	2.39	7.70	1.06	17,389	399
I-7	20,747	3.57	15.23	3.31	5.37	58.82	324,972	308
I-8	20,747	6.54	14.14	6.36	4.25	30.95	308,130	309
H-1	31,436	0.00	4.87	3.26	10.00	1.18	6,972	244
H-2	31,436	0.16	4.65	3.50	10.00	1.05	7,273	219
H-3	31,436	0.00	2.91	2.00	10.00	1.42	6,396	105
H-4	31,436	0.00	2.65	3.98	10.00	1.21	5,243	96
H-5	31,750	0.00	8.60	3.82	6.93	0.27	1,335	458
H-6	31,750	0.00	3.49	2.40	8.25	0.40	2,194	461

This table presents the collected data for the variables used in the model development in this study.

## Identification of variables influencing retroreflectivity of road markings

The independent variables—damage area, measured area, and damage ratio of pavement lane markings; fatigue cracking ratio; rutting depth; IRI and SPI of pavement conditions; and AADT—were selected to identify their effect on the retroreflectivity of pavement lane markings as the dependent variable. However, influencing factors such as traffic, weather, and season were selected in previous studies. Seasonal and weather conditions were excluded in this study because the pavement markings were subject to the same conditions within Seoul City. Pavement conditions beneath road markings can affect the condition of the markings themselves. Therefore, the measured conditions of crack ratio, rutting depth, IRI, and SPI were selected as independent variables. Moreover, since both pavement and road markings are damaged by traffic-related factors such as vehicle weight and friction, AADT was assumed to be an independent variable.

**Figure 4.**
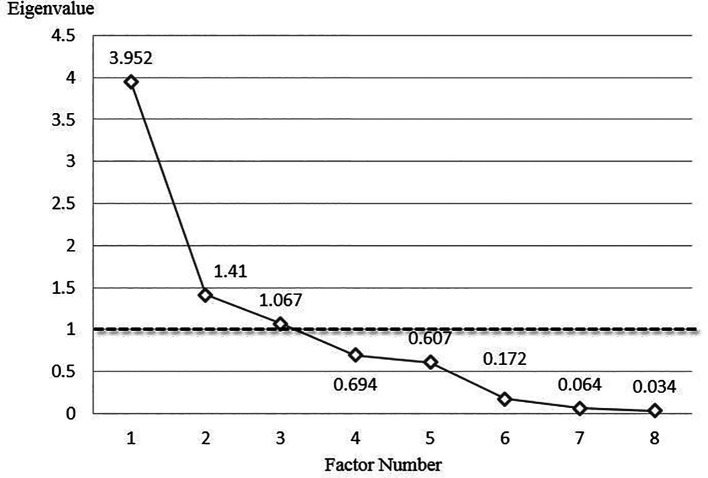
Scree Diagram Result from Principal Component Analysis. A scree diagram was derived from Principal Component Analysis (PCA). Statistically, the number of clusters is determined at the point where the eigenvalue falls below 1. Consequently, three clusters were deemed appropriate in this study, and the result is presented in Figure 4.

Factor Analysis (FA), Principal Component Analysis (PCA), and multi-regression analysis were applied to determine the influence of the independent variables on the dependent variable of retroreflectivity. The Maximum Likelihood Method (MLM) was applied to the FA, assuming that the multivariate data followed a normal distribution. FA was used to identify the clustering of variables by analyzing the scree diagram to determine the number of clusters based on eigenvalue variation. Eigenvalues explain the variance in the major factors. After approximating the number of clusters, PCA was performed to determine whether new clusters could be formed by constructing more influential factors from the classification of the independent variables. The linear effects of individual independent variables on the dependent variable were confirmed through multi-regression analysis. In other words, the clustering and classification capability, the basic statistics of the variables, and a preliminary review of the influential factors were verified using FA and PCA. Multi-regression analysis was then used to assess the effectiveness of the independent variables on retroreflectivity and to develop a linear estimation model. Table 4 presents the descriptive statistics of the variables. The scree diagram results from the PCA, based on the eigenvalue criterion of 1, are shown in Figure 4.

**Table 4.** Descriptive Statistics of Variables.

Variable	Average	Standard deviation	Correlation	Etc.
AADT (vehicles/day)	23,097.10	6,485.05	0.220	Kaiser-Meyer-Olkin measure of sampling adequacy: 0.545 Bartlett’s test of sphericity (significance): 0.000
Crack Ratio (%)	3.26	5.34	-0.144	
Rutting depth (mm)	7.14	4.21	0.213	
IRI (m/km)	3.96	2.27	-0.083	
SPI	6.37	2.54	-0.016	
Damage ratio (%)	11.85	17.97	0.080	
Pavement marking area (mm ^2^)	1.05E+6	7.00E+5	-0.015	
Damage area (mm ^2^)	115,772.65	1.97E+5	0.096	
Retroreflectivity (mcd/lux·m ^2^)	245.65	122.28	1.000	

This table shows the results of basic statistical analysis of the variables.

As shown in Figure 4, the PCA and FA analyses were deemed appropriate according to the Kaiser-Meyer-Olkin (KMO) value and the significance level of the p-value. In addition, the standard deviations of AADT, the measured pavement marking area, and the damaged area of road markings were higher than those of the other variables. This indicates that the variables need to be normalized to control for unit or dimensional differences. Furthermore, variables excluding retroreflectivity could be clustered or controlled using three factors derived from FA and PCA. This was considered reasonable due to the statistical explanation rate of 74.32% when three factors were selected.

Multi-regression analysis, based on the results of the basic statistical analysis, FA, and PCA, was performed to verify the linear effect of the independent variables on the dependent variable. Variables with high variation were transformed using logarithmic values to control for standard deviation. The results of the multi-regression analysis are presented in Tables 5 and 6.

**Table 5.** Statistical Results of Multi-Regression Analysis for Influence Variable Verification.

$R^{2}$	Standard error of the estimate	Statistical variation			Durbin-Watson	Etc.
		$R^{2}$	F	Significance probability F		
0.719	99.18	0.719	2.95	0.021	1.631	

This table presents the results of multi-regression analysis conducted to evaluate the linear influence of independent variables on the dependent variable.

**Table 6.** Constructed Model Results of Multi-Linear Regression Analysis.

	Standardized coefficient		Standardized coefficient	t	Significance probability	Collinear statistics		Etc.
	B	Standardized error	β			tolerance	VIF	
Intercept	-1,457.82	1,173.25		-1.24	0.23			
AADT (vehicles/day)	394.26	225.81	0.38	1.75	0.05	0.46	2.17	
Crack ratio (%)	-14.54	7.20	-0.63	-2.02	0.05	0.22	4.51	
Rutting depth (mm)	14.94	12.68	0.52	1.18	0.25	0.12	8.70	
IRI (m/km)	-34.03	14.13	-0.63	-2.41	0.02	0.32	3.13	
SPI	-50.53	25.08	-1.05	-2.02	0.05	0.08	12.37	
Damage ratio (%)	1.46	2.57	0.21	0.57	0.58	0.15	6.48	
Pavement marking area ( ${\mathrm{mm}}^{2}$ )	157.67	95.53	0.30	1.65	0.11	0.66	1.52	
Damage area ( ${\mathrm{mm}}^{2}$ )	-124.92	85.53	-0.74	-1.91	0.06	0.15	6.83	

This table shows the results of multi-regression analysis conducted to identify the influence of independent variables on the dependent variable, retroreflectivity. Traffic volume, pavement marking damage rate, and SPI were selected as influencing factors.

As listed in Tables 5 and 6, the analysis results showed that the major factor affecting retroreflectivity was the SPI value. However, a statistically significant negative relationship between the variables was found. Moreover, IRI and crack ratio values were also shown to have significant influence. The presence of multicollinearity among the variables was confirmed based on the Variance Inflation Factor (VIF) and tolerance values. It is because the SPI is determined by pavement conditions such as crack ratio, rutting depth, and IRI. However, the pavement marking conditions—damage area, measured area, and damage ratio—were not found to be highly effective, and the damage ratio of pavement markings is statistically difficult to identify as a major influencing factor.

The statistical results indicate that pavement conditions affect retroreflectivity because the SPI value used for pavement maintenance evaluation reflects general damage conditions. Traffic conditions, represented by AADT, also negatively impact retroreflectivity, as road markings and beads are worn by vehicles in pavement engineering. Among the conditions regarding road markings, the damage area can physically have the largest effect on retroreflectivity. Therefore, the variables AADT, SPI, and damage area of pavement markings were selected as effective influencing factors.

## Development of a predictive model, a degradation model, and an evaluation model with retroreflectivity of road lane markings

The objective of this study is to develop a retroreflectivity prediction model using a measurement vehicle to detect pavement damage and road marking conditions. This means that the conditions of the pavement and road markings can be inspected simply using this measurement method, and the retroreflectivity can be easily determined using the measured data. The performance degradation prediction model with pavement marking age and the performance evaluation model can be utilized by applying the retroreflectivity prediction model. To achieve these objectives, the measured and collected data were used to construct models based on the dependent variable of retroreflectivity, and the GRG algorithm was utilized to minimize the Root Mean Squared Error (RMSE) between the measurements and predictions.

In the first step of developing the retroreflectivity prediction model, the regression approach for determining the coefficients in the model was applied to the measured data using a vehicle to measure the damage to pavement and road markings and the AADT from TOPIS administered by Seoul City. While developing the model, the first linear to *n*-polynomial nonlinear model was constructed based on the assumption that individual independent variables can nonlinearly affect the dependent variable. The errors and tendencies of the models were compared to determine the optimal model. The basic function of the *n*-polynomial nonlinear regression model is expressed as Eq. (3):

$$R_{L,\text{predict}}=\beta_{0}+\sum_{i=1}^{n}\beta_{\text{AADT},i}{\text{AADT}}^{i}+\beta_{\mathrm{SPI},i}{\mathrm{SPI}}^{i}+\beta_{\mathrm{DA},i}{\mathrm{DA}}^{i}$$ (2)

where β and *i* represent the regression coefficient and the number of the *n*-polynomial function from 1 to *n.* The AADT, SPI, and DA (Damage Area) are the independent variables.

**Figure 5.**
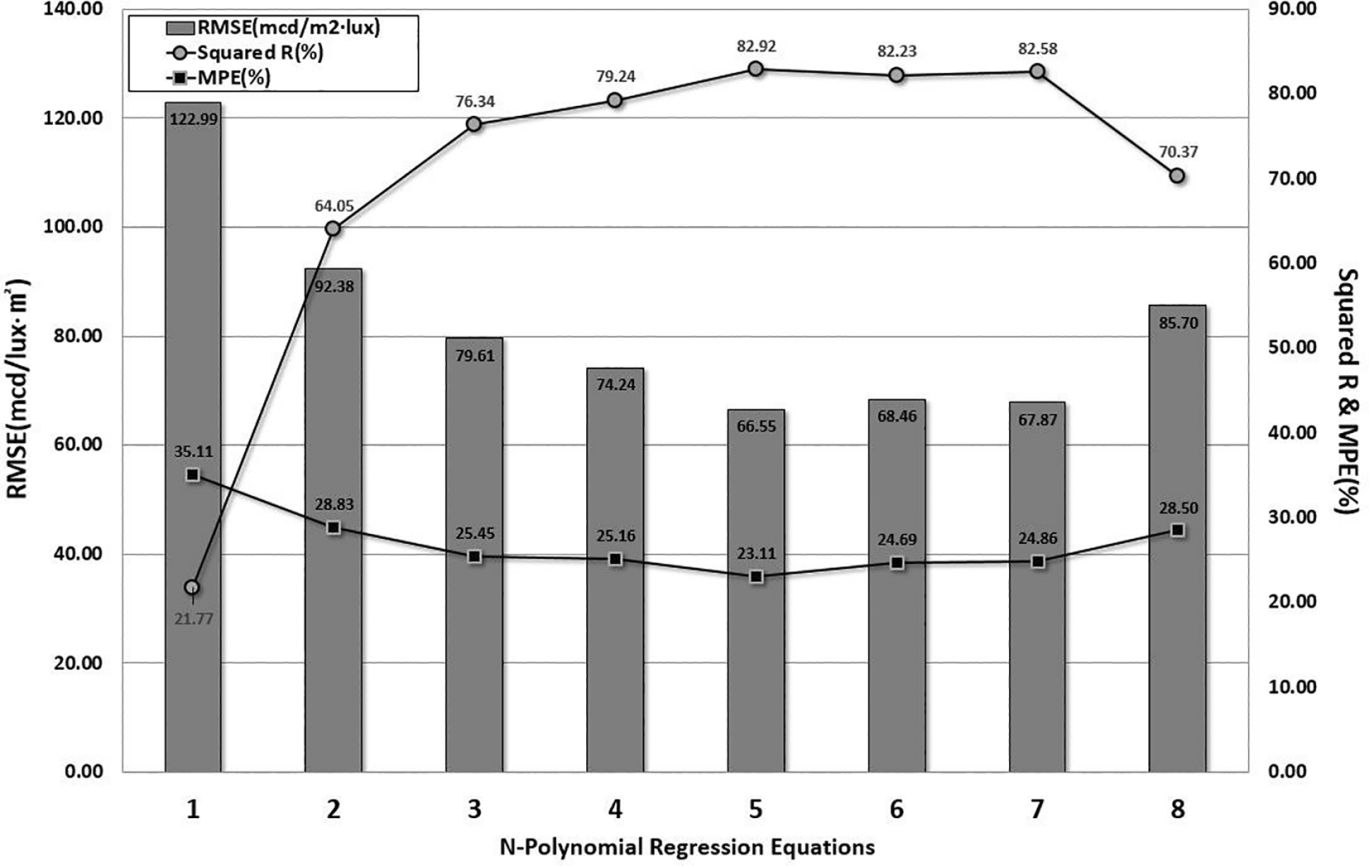
RMSE, MPE, and Determination Coefficient from Regression Analysis. This study applied the n-polynomial regression analysis method. Model evaluation was discontinued when Root Mean Sqaured Error (RMSE) and Mean Percentage of Error (MPE) increased sharply, and the determination coefficient decreased significantly. The evaluation was stopped at the 8th-order polynomial model, and the 5th-order polynomial model was selected as the optimanl prediction model due to its lowest RMSE and MPE and highest Determination Coefficient.

In this research, *n* is set to stop when the RMSE and MPE dramatically increase, or the value of the determination coefficient significantly decreases, based on engineering judgment. The optimum model with the lowest RMSE and MPE and the highest value of R-squared was determined by comparing the results. The comparison results are shown in Figure 5.

**Figure 6.**
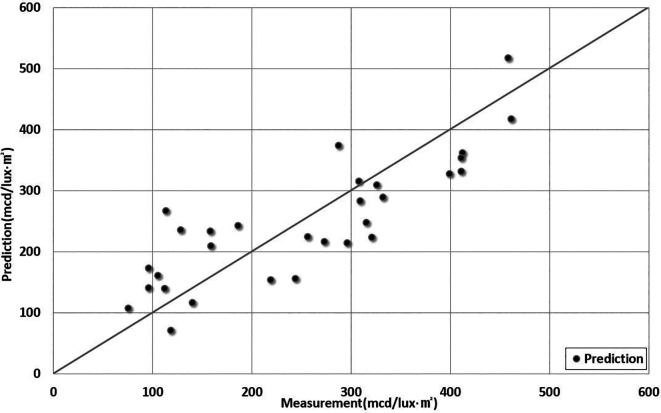
Comparison Between Predictions and Measurements. This figure compares the predicted retroreflectivity results obtained from the 5th-order polynomial regression model developed in this study with the actual measured values.

The first-order linear regression model exhibited the highest RMSE and MPE values. In addition, the determination coefficient of the 8-polynomial nonlinear regression model decreased dramatically. There were minor differences in the RMSE and MPE between the 5th-order model and the 7th-order model. Therefore, the 5-polynomial regression model indicated the lowest RMSE and MPE values, and the highest determination coefficient was obtained for the retroreflectivity prediction model. The regression coefficients determined using the optimal model are listed in Table 7. The predictions of the measurements are shown in Figure 6.

**Table 7.** Regression Coefficients Determined for the Retroreflectivity Prediction Model.

Coef.	$\beta_{0}\;$ (Intercept)	$\beta_{\text{AADT},1}$	$\beta_{\mathrm{DM},1}$	$\beta_{\mathrm{SPI},1}$	$\beta_{\text{AADT},2}$	$\beta_{\mathrm{DM},2}$	$\beta_{\mathrm{SPI},2}$	$\beta_{\text{AADT},3}$
Value	-15,806.7	0.0034	158.0606	3.9859	1,331.78	-54.3931	10.1179	19.8934
Coef.	$\beta_{\mathrm{DM},3}$	$\beta_{\mathrm{SPI},3}$	$\beta_{\text{AADT},4}$	$\beta_{\mathrm{DM},4}$	$\beta_{\mathrm{SPI},4}$	$\beta_{\text{AADT},5}$	$\beta_{\mathrm{DM},5}$	$\beta_{\mathrm{SPI},5}$
Value	-0.0131	-0.1641	-0.7934	-0.0053	-0.1991	-6.6579	0.1144	0.0100

Using the selected influencing variables, the model was developed via n-polynomial regression analysis. The 5th-order polynomial regression model was determined as the optimal model. The coefficients of the optimal model are presented in this table.

Approval for the installation of pavement markings in Seoul City is granted when the performance of the retroreflectivity within a week after installation completion is higher than the standard of 240 mcd/lux·m ^2^ according to the completion inspection, and the performance is actually evaluated. In this regard, records of the completion inspection of the installation were utilized to develop the degradation prediction model with the age of road markings on the research road sections.

In developing the degradation model, previous research dealing with initial retroreflectivity was intensively reviewed because this study addresses the initial values at the completion inspections after the work is completed. Previous studies investigated the influencing factors affecting the service life and performance degradation of road markings ( Zhao et al., 2023). In previous research, the effective variables associated with service life were traffic volume, initial retroreflectivity, lane marking width, and color. This study is similar to previous ones in that performance degradation can affect the service life of road markings. However, weather conditions were not selected as independent variables because the spatial range of this research was restricted to Seoul City. Pavement marking conditions such as width, color, and shape were excluded because of the objective of this research regarding pavement lane markings. Therefore, the available variables—measured retroreflectivity, initial retroreflectivity at completion inspection, age in service, and traffic volume (AADT)—were utilized to develop the prediction model. In addition, various model functions used in previous studies, such as logarithmic, linear regression, multiple linear regression, exponential, and Markov chain models, were considered. In this study, the logarithmic and multilinear regression functions were selected to determine the optimum model by comparing the models.

**Figure 7.**
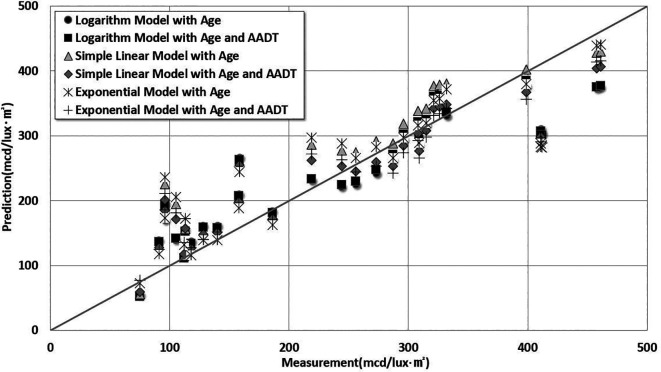
Comparative Analysis Between Estimation Model Predictions and Measurements. This study analyzed the degradation of pavement marking retroreflectivity according to marking life and traffic volume by comparing logarithmic, simple linear, and exponential models. The predicted results were compared with the actual lifespan of pavement markings.

Furthermore, the damage and behavior of thermoplastic road marking materials with viscoelasticity are similar to those of asphalt pavement materials used in material engineering. However, rutting deformation in the road does not occur or is negligible because the installation of road markings is a thin coating method. Thus, the behavior of road markings can be regarded as similar to that of the pavement ( Henry et al., 1981). Pavement conditions for management implementation and service life repair can be exponentially expressed by performance degradation ( Al-Mansour et al., 2022). Based on engineering judgment, an exponential function is introduced for developing the performance degradation model of retroreflectivity. The performance degradation models and error results are listed in Table 8. The comparison results between the predictions and measurements are shown in Figure 7.

**Table 8.** Research Models and Analysis Results for Estimating Reduction of Retroreflectivity.

Model	Equation	Independent variables	Analysis Results		
			RMSE	$R^{2}$	MPE
Logarithm	$R_{L,\text{estimation}}=R_{\text{initial}}-\alpha \;\log\left(x_{1}\right)$	Age	54.25	0.68	0.37
	$R_{L,\text{estimation}}=R_{\text{initial}}-\alpha \log\left(x_{1}\right)-\beta \;\log\left(x_{2}\right)$	Age, AADT	54.02	0.91	0.17
Simple linear model	$R_{L,\text{estimation}}=R_{\text{initial}}-\alpha \;x_{1}$	Age	59.32	0.53	0.32
	$R_{L,\text{estimation}}=R_{\text{initial}}-\alpha \;x_{1}-\beta \;x_{2}$	Age, AADT	54.62	0.91	0.16
Exponential model	$R_{L,\text{estimation}}=R_{\text{initial}}-\alpha \;\exp\left(x_{1}\right)$	Age	60.04	0.56	0.33
	$R_{L,\text{estimation}}=R_{\text{initial}}-\alpha \exp\left(x_{1}\right)-\beta \;\exp\left(x_{2}\right)$	Age, AADT	56.98	0.90	0.17

To estimate the performance degradation of pavement markings based on retroreflectivity, this study evaluated logarithmic, simple linear, and exponential models. The independent variables used were initial retroreflectivity after installation, pavement marking life, and traffic volume. The purpose was to identify the most suitable model, and the results are presented in this table.

As shown in Figure 7, the logarithmic prediction model for retroreflectivity degradation based on the age of lane markings and AADT had the lowest RMSE of 54.02 mcd/lux·m ^2^ and the highest determination coefficient of 91%. The simple linear regression model including age and AADT showed the lowest MPE (16%).

Another objective of this study was to develop a maintenance index model using retroreflectivity and damage severity. In Seoul City, the maintenance index model for evaluating road marking performance is insufficient for determining maintenance and repair work. Therefore, the determination and implementation of maintenance and repair work rely only on the standards of retroreflectivity, damage severity, and civil complaints. The maintenance index model can be utilized for budget formulation regarding repair work and reinstallation, as well as their maintenance. As mentioned earlier, road markings provide visibility to drivers. The damage condition can affect visibility during the daytime, and retroreflectivity performance can be directly connected with visibility at night or under foggy and rainy conditions. Therefore, the performance regarding damage and retroreflectivity can be major factors in developing the maintenance index model.

In this regard, a performance evaluation model was developed for the maintenance index using retroreflectivity and damage. In Seoul Metropolitan City, pavement conditions are managed and maintained using SPI scores ranging from 0 to 10. Considering this condition, the model development was targeted at a maximum index score of 10. This implies that the conditions for the pavement and road markings can be administered using similar approaches. Road markings have absolute maximum performance and function once the installation is completed. In the best condition immediately after installation or repair work, the road markings have the lowest damage and highest retroreflectivity. Therefore, the crack ratio and percentage of retroreflectivity performance degradation can be utilized to construct a maintenance index model. This can be expressed by (3).

$$\text{SRMI}={\text{SRMI}}_{\text{retroreflectivity}}+{\text{SRMI}}_{\text{CrackRatio}}=5\frac{R_{L,\text{present}}}{R_{L,\text{initial}}}+5\frac{\left(100-\mathrm{CR}\right)}{100}$$ (3)

where SRMI, ${\text{SRMI}}_{\text{retroreflectivity}}$ , ${\text{SRMI}}_{\text{Crack Ratio}}$ , $R_{L,\text{Present}}$ , $\;R_{L,\text{initial}}$ and CR are the Seoul Road Marking Index, Seoul Road Marking Index for the retroreflectivity degradation ratio, Seoul Road Marking Index for the crack ratio, present retroreflectivity, initial retroreflectivity, and crack ratio, respectively.

The maintenance evaluation index assumes that the effectiveness of luminance and damage act equivalently on the index. However, controlled effectiveness conditions can be dissimilarly required, and the maintenance index model can be applied differently to road conditions. Therefore, another maintenance index model for evaluating the performance of road markings, which can be utilized under varying road conditions, is expressed in (4).

$$\begin{array}{c} \text{SRMI}=k_{1}{\text{SRMI}}_{\text{retroreflectivity}}+k_{2}{\text{SRMI}}_{\text{CrackRatio}}\\ =5k_{1}\frac{R_{L,\text{present}}}{R_{L,\text{initial}}}+5k_{2}\frac{\left(100-\mathrm{CR}\right)}{100}\\ =5k_{1}\frac{R_{L,\text{present}}}{R_{L,\text{initial}}}+5\left(2-k_{1}\right)\frac{\left(100-\mathrm{CR}\right)}{100} \end{array}$$ (4)

where $k_{1}$ and $k_{2}$ are the consideration coefficients for the road conditions. The value of $k_{1}$ ranges from 0 to 2, and the relationship between $k_{1}$ and $k_{2}=2-k_{1}$ .

**Figure 8.**
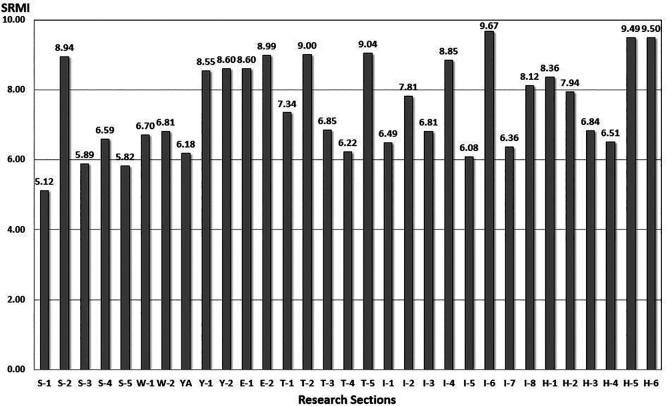
Performance Evaluation Score from SRMI model. The Seoul Road Marking Index (SRMI) model developed in this study was applied to evaluate current road conditions. This figure presents the evaluation results.

The maintenance index model implies that the value of $k_{1}$ can be determined at the maximum value of 2 if the road condition requires the luminance performance of road markings, as in tunnels or box structures. In such cases, the maintenance index is determined only by the retroreflectivity performance under damaged conditions. Conversely, if maintenance work is required solely based on damage severity, the value of $k_{1}\;$ can be set to 0. This implies that maintenance is administered only according to the damage condition. However, the values of $k_{1}$ and $k_{2}$ should be effectively determined by the maintenance office or administrators, regardless of whether the road is in an extreme condition. It is advisable that the values of $k_{1}$ and $k_{2}$ be set to 1 under general conditions. The evaluation results of the maintenance index model after applying the model to this research section are shown in Figure 8.

From the result in Figure 8, road markings are managed by road sections or offices. The implementation of maintenance and repair work can be determined using this model. In addition, the model can be utilized for future budget formulations and road-marking management plans in Seoul City.

## Conclusion

Because the measurements of luminance performance and damage to road markings are implemented separately in Seoul City, errors related to time-based, locational, and environmental conditions can occur. Moreover, separate measurements have temporal and economic limitations and can lead to civil complaints. To address these issues, a model using a vehicle for detecting damage to road markings was developed in this research. The 5-polynomial regression model was determined to be the optimal prediction model for the performance prediction of retroreflectivity. The developed model was constructed using SPI, the damaged area of the road marking, and AADT, allowing easy prediction of retroreflectivity simply by using a vehicle to measure pavement conditions as well as the crack condition of the road markings. The traffic information for AADT can be acquired from the national information-sharing system of TOPIS. This indicates that the model has high utility and applicability. Many potential advantages are expected in terms of time, cost, safety, etc. because the model can predict retroreflectivity more easily and quickly. In addition, a degradation model of luminance performance with age in service was developed. In this study, the error and trend results from logarithmic, simple linear, and exponential models using age and traffic conditions were compared to determine the optimal model. The logarithmic performance degradation model for retroreflectivity, based on age and AADT, was selected based on the lowest RMSE and highest determination coefficient. This model can estimate performance degradation, which makes it suitable for use in management, budget estimation, and repair work planning. A performance evaluation model with a maintenance index was developed using the SRMI. The model is similar to the SPI used in pavement management and was developed due to the absence of management and evaluation models for determining the implementation of maintenance and repair work in Seoul City. It can be applied to various road conditions, and performance evaluations can be conducted using the developed models. The weights of the model developed in this study can be determined for maintenance purposes based on the decisions of the relevant road management authorities. Additional research on methods for determining these weights will be conducted in the future.

We expect that maintenance standards and legislation can be established, and that budget formulation and maintenance planning can be guided by the utilization of this model. Moreover, we expect that the developed models for predicting retroreflectivity, luminance performance degradation, and performance evaluation can be applied in Seoul City, as they allow for easier and faster prediction and evaluation of road marking performance. Finally, future studies will aim to conduct a more detailed analysis covering the overall road conditions in Seoul and various road sections. In addition, techniques such as ridge regression and principal component regression will be employed to ensure the stability of coefficient interpretation in the proposed model.

## Data Availability

Figures: Development of Predictive and Evaluation Models for the Retroreflectivity Performance of Pavement Lane Markings. https://doi.org/10.57760/sciencedb.28828. ( Kim, 2025). This research contains the following underlying data:
•
Figure 4_Data (Raw data of scree diagram result) •
Figure 5_Data (Raw data of RMSE, MPE, and Determination Coefficient from Regression Analysis) •
Figure 6_Data (Raw data of comparison between predictions and measurements) •
Figure 7_Data (Raw data of comparative analysis between estimation model predictions and measurements) •
Figure 8_Data (Raw data of performance evaluation score from SRMI mode) Data are available under the terms of the Creative Commons Attribution 4.0 International license (CC-BY 4.0).
