# Supplementary material for: Development of predictive and evaluation models for the retro reflectivity performance of pavement lane markings
Source: F1000Res. 2025 Oct 6;14:907. Originally published 2025 Sep 11. [Version 2] doi: 10.12688/f1000research.169127.2 (PMC12579311; doi:10.12688/f1000research.169127.2)
Supplement: Peer review history

# Reviewer report: Reviewer response for version 2

**DOI:** 10.5256/f1000research.188988.r422202

**Published:** 2025-10-31

**Authors:** Dario Babic

**Affiliations:** 1 University of Zagreb Faculty of Transport and Traffic Sciences, Zagreb, Croatia

The main aim of the study is to develop a predictive and evaluation model for the retroreflectivity of road markings in Seoul. In the study, authors integrated data from different sources – retroreflectivity measurements, pavement data from pavement damage detection and traffic data. The study provides new insights into retroreflectivity modelling and provides practical solutions to road markings maintenance.

Is the work clearly and accurately presented and does it cite the current literature?

Yes

If applicable, is the statistical analysis and its interpretation appropriate?

Yes

Are all the source data underlying the results available to ensure full reproducibility?

Yes

Is the study design appropriate and is the work technically sound?

Yes

Are the conclusions drawn adequately supported by the results?

Yes

Are sufficient details of methods and analysis provided to allow replication by others?

Yes

Reviewer Expertise:

road safety; road asset management; traffic control devices

I confirm that I have read this submission and believe that I have an appropriate level of expertise to confirm that it is of an acceptable scientific standard.

# Reviewer report: Reviewer response for version 2

**DOI:** 10.5256/f1000research.188988.r420553

**Published:** 2025-10-14

**Authors:** Zhihao Xu

**Affiliations:** 1 Institute of Ubiquitous Networks & Urban Computing, Qingdao University, Qingdao, China

Approved. I have no furthermore questions,

Is the work clearly and accurately presented and does it cite the current literature?

Yes

If applicable, is the statistical analysis and its interpretation appropriate?

Yes

Are all the source data underlying the results available to ensure full reproducibility?

Yes

Is the study design appropriate and is the work technically sound?

Yes

Are the conclusions drawn adequately supported by the results?

Yes

Are sufficient details of methods and analysis provided to allow replication by others?

Yes

Reviewer Expertise:

ITS; Machine learning

I confirm that I have read this submission and believe that I have an appropriate level of expertise to confirm that it is of an acceptable scientific standard.

# Reviewer report: Reviewer response for version 1

**DOI:** 10.5256/f1000research.186413.r417659

**Published:** 2025-09-27

**Authors:** Zhihao Xu

**Affiliations:** 1 Institute of Ubiquitous Networks & Urban Computing, Qingdao University, Qingdao, China

This study focuses on developing a predictive and evaluation model for the retro reflectivity of road markings in Seoul, addressing clear engineering applications and practical management needs. The authors innovatively integrate data from pavement damage detection vehicles with traffic volume to construct a retro reflectivity prediction model, thereby avoiding the spatio-temporal inconsistencies and safety risks inherent in traditional retro reflectivity measurements. This method offers an efficient, low-cost alternative for urban road marking maintenance, demonstrating strong practical value and potential for widespread adoption.

 1. The authors employed data on pavement conditions, lane marking damage, and retro reflectivity from 31 road sections, combined with AADT, to conduct factor analysis, principal component analysis, and multiple regression analysis, demonstrating a clear technical approach. Notably, the authors employed Mask R-CNN for image segmentation of damaged marking areas, combined with binarization processing—an advanced and reproducible approach. However, the relatively limited sample size (n=31) and the fact that all data originated from Seoul's urban area may constrain the model's generalization capability.

 2. In the retro reflectivity prediction model, the authors compared polynomial regression models ranging from first to eighth order, ultimately selecting the fifth-order model as the optimal solution due to its lowest RMSE and MPE alongside the highest R². Key independent variables included SPI, AADT, and damaged area, with SPI exhibiting a negative coefficient consistent with engineering practice. However, multicollinearity exists among some variables in the model. Although addressed in the paper, this may still affect the stability of coefficient interpretation. It is recommended to consider using ridge regression or principal component regression to mitigate this issue.

 3. Based on the service life of road markings and AADT, the authors compared three degradation models: logarithmic, linear, and exponential. The logarithmic model was ultimately selected as the optimal model, achieving an R² of 0.91 and an RMSE of 54.02. This model effectively predicts the temporal decay trend of retro reflectivity and is suitable for maintenance planning. Additionally, the proposed Seoul Road Marking Index (SRMI) combines retro reflectivity retention rate with damage rate, incorporating weighting coefficients k₁ and k₂ to adapt to varying road conditions. Its structure is both rational and flexible. However, the SRMI's assumption of equal weighting for both components lacks empirical support. Further calibration of weight distribution through driver visual perception experiments is recommended.

 4. This study excels in method selection, model construction, and engineering translation, demonstrating particular strengths in integrating multi-source data, applying AI image processing, and developing practical evaluation indices. However, the paper does not address the model's applicability under extreme weather conditions; supplementary discussion is recommended.

Is the work clearly and accurately presented and does it cite the current literature?

Yes

If applicable, is the statistical analysis and its interpretation appropriate?

Yes

Are all the source data underlying the results available to ensure full reproducibility?

Yes

Is the study design appropriate and is the work technically sound?

Yes

Are the conclusions drawn adequately supported by the results?

Yes

Are sufficient details of methods and analysis provided to allow replication by others?

Yes

Reviewer Expertise:

ITS; Machine learning

I confirm that I have read this submission and believe that I have an appropriate level of expertise to confirm that it is of an acceptable scientific standard, however I have significant reservations, as outlined above.

# Response

**Published:** 2025-09-27

**Authors:** Dowan Kim

**Affiliations:** Road & Airport Dept, kunhwa Engineerin & Consulting, Seoul, South Korea

Before responding to the reviewer’s comments, I would like to express my sincere gratitude for your interest in and thoughtful evaluation of our study. We have carefully reviewed your feedback and have prepared detailed and sincere responses to each of your comments.

 To begin, I would like to provide some context for our study. This research represents an initial effort to evaluate and predict the retroreflectivity performance of road lane markings and to overcome limitations associated with traditional investigation methods. We plan to conduct further follow-up studies to expand upon the findings of this work. In this regard, the comments and suggestions you have provided are extremely valuable and will be highly beneficial for the continuation and enhancement of our research. We greatly appreciate your insights and respectfully ask you to review our responses below.

 1.

 As you rightly pointed out, this study was conducted based on data from 31 sections across 8 city roads within Seoul. We acknowledge that this sample size limits the generalizability of the results to other road types such as expressways, main roads, or local roads across the city. This is indeed a limitation related to the representativeness of the road sample. Accordingly, **we will revise the Conclusion section to explicitly mention this limitation**, and in future studies, we will aim to include a larger and more diverse sample of roads across Seoul.

 2.

 As mentioned above, we plan to expand future studies by incorporating a larger number of road samples under varying conditions. In alignment with your suggestion, we will integrate ridge regression and principal component regression in our future models to address multicollinearity and improve model robustness. We appreciate your guidance on this matter, and **we will also note this direction for future research in the Conclusion section**.

 3.

 We agree with your insightful comment regarding the weighting coefficients in the Seoul Road Marking Index (SRMI). Specifically, as you indicated, coefficient k₁ reflects the necessity of retroreflectivity depending on road conditions, while coefficient k₂ reflects the importance of visibility related to the level of lane marking damage from the driver’s perspective. These coefficients are typically determined by local road management authorities based on practical considerations. Therefore, in this study, we adopted a generalized approach and did not attempt to define these coefficients based on a specific administrative procedure. And, in the chapter *“Development of a predictive model, a degradation model, and an evaluation model with retroreflectivity of road lane markings,”* we have mentioned that these coefficients can vary depending on decisions made by road management authorities. We also agree that further empirical validation, possibly through visual perception studies involving drivers, should be pursued in future research. **We will reflect this point in the Conclusion section of the paper.**

 4.

 As you correctly noted, this study does not consider extreme weather conditions. This is primarily due to our focus on overcoming the limitations of current measurement methodologies through an investigation-based approach. Nonetheless, we fully agree that weather—particularly extreme conditions—can significantly impact the service life and performance of lane markings.

 While it was beyond the scope of this particular study, we recognize the importance of such factors, and we plan to explore them either in future extensions of this research or through separate studies that incorporate variables such as traffic volume, weather conditions, and lane marking types. Thank you again for providing a valuable research direction for us to consider.

 Once again, we sincerely thank you for your interest, constructive comments, and thorough review. Your feedback has greatly contributed to the improvement and future development of our research.
